# Fecal β-glucuronidase activity differs between hematopoietic cell and kidney transplantation and a possible mechanism for disparate dose requirements

**DOI:** 10.1080/19490976.2022.2108279

**Published:** 2022-08-03

**Authors:** Mohammad Haneef Khan, Guillaume C. Onyeaghala, Armin Rashidi, Shernan G. Holtan, Alexander Khoruts, Ajay Israni, Pamala A. Jacobson, Christopher Staley

**Affiliations:** a Department of Surgery, University of Minnesota Medical School, Minneapolis, MN, United States; b Hennepin Healthcare Research Institute, Minneapolis, MN, United States; c Hematology, Oncology, and Transplantation, University of Minnesota Medical School, Minneapolis, MN, United States; d Gastroenterology, Department of Medicine, University of Minnesota Medical School, Minneapolis, MN, United States; e Nephrology, Department of Medicine, University of Minnesota Medical School, Minneapolis, MN, United States; f Experimental and Clinical Pharmacology, University of Minnesota College of Pharmacy, Minneapolis, MN, United States

**Keywords:** Beta-glucuronidase, immunosuppression, intestinal microbiota, mycophenolate, MMF, transplantation

## Abstract

The intestinal microbiota produces β-glucuronidase that plays an essential role in the metabolism of the immunosuppressant mycophenolate mofetil (MMF). This drug is commonly used in organ and hematopoietic cell transplantation (HCT), with variations in dosing across transplant types. We hypothesized that β-glucuronidase activity differs between transplant types, which may account for differences in dosing requirements. We evaluated fecal β-glucuronidase activity in patients receiving MMF post-allogeneic HCT and post-kidney transplant. Kidney transplant patients had significantly greater β-glucuronidase activity (8.48 ± 6.21 nmol/hr/g) than HCT patients (3.50 ± 3.29 nmol/hr/g; *P* = .001). Microbially mediated β-glucuronidase activity may be a critical determinant in the amount of mycophenolate entering the systemic circulation and an important factor to consider for precision dosing of MMF.

## To the editor

The intestinal microbiota is associated with human health and performs many functions for the body including protective, metabolic, and structural functions.^1^ Antibiotic and immunosuppressive treatments, however, adversely affect the composition and functions of the microbiota.^2–4^ Recently, the microbiota has received attention as a collection of agents that can affect the efficacy of drugs, including immunosuppressants (IS), by altering normal metabolism and/or transport, or by facilitating drug bioaccumulation in cells.^4–8^ Mycophenolate mofetil (MMF) is an IS prodrug that is widely used in combination with tacrolimus (TAC) for post-allogeneic hematopoietic cell transplantation (HCT) and kidney transplantation. The active form of MMF, mycophenolic acid (MPA), inhibits the proliferation of T and B lymphocytes thereby reducing the risk of graft-*versus*-host disease (GVHD), improves stem cell engraftment after HCT, and reduces the risk of allograft rejection after kidney transplant.^9^ Blood MPA concentrations are influenced by enterohepatic recycling (EHR) of MPA due to the de-glucuronidation of MPA glucuronide (MPAG, major inactive metabolite of MMF) by microbial β-glucuronidase post-biliary excretion.^4,10^ We recently showed that therapeutic concentrations of MPA in the blood of HCT patients were associated with the microbiome composition measured in their fecal samples, although the mechanism for this finding remains unknown.^4^ We observed that individuals with therapeutic MPA concentrations were more likely to have β-glucuronidase-producing bacteria in their stool and significantly greater EHR and reformation of MPA.

Despite the importance of MMF in facilitating donor engraftment and preventing GVHD, 50% of patients suffer from GVHD and associated non-relapse mortality,^4,11^ and 5–10% of kidney transplant recipients with have an acute rejection event leading to a higher risk of kidney graft loss.^12^ Variability in MPA plasma concentrations among patients affects the potential for adverse clinical outcomes. Lower concentrations increase the risk of GVHD and rejection, while higher concentrations are associated with toxicity.^10,13,14^ There is a well-known difference in the doses of MMF needed to achieve therapeutic concentrations in HCT and kidney transplant recipients. Lower MPA exposure is observed in HCT recipients compared to kidney transplant recipients receiving a similar MMF dose.^15–17^ Reasons for this difference are not understood but may be due to lower β-glucuronidase activity in the stool of HCT recipients, resulting in lower enterohepatic recirculation and reformation of MPA, lower MPA exposure in the blood, and the need for higher MMF doses. Understanding microbially mediated processes that affect MPA exposure is required to inform precision MMF dosing and for other drugs that undergo β-glucuronidase-mediated enterohepatic recirculation (e.g. irinotecan).^18^

**Figure 1.**
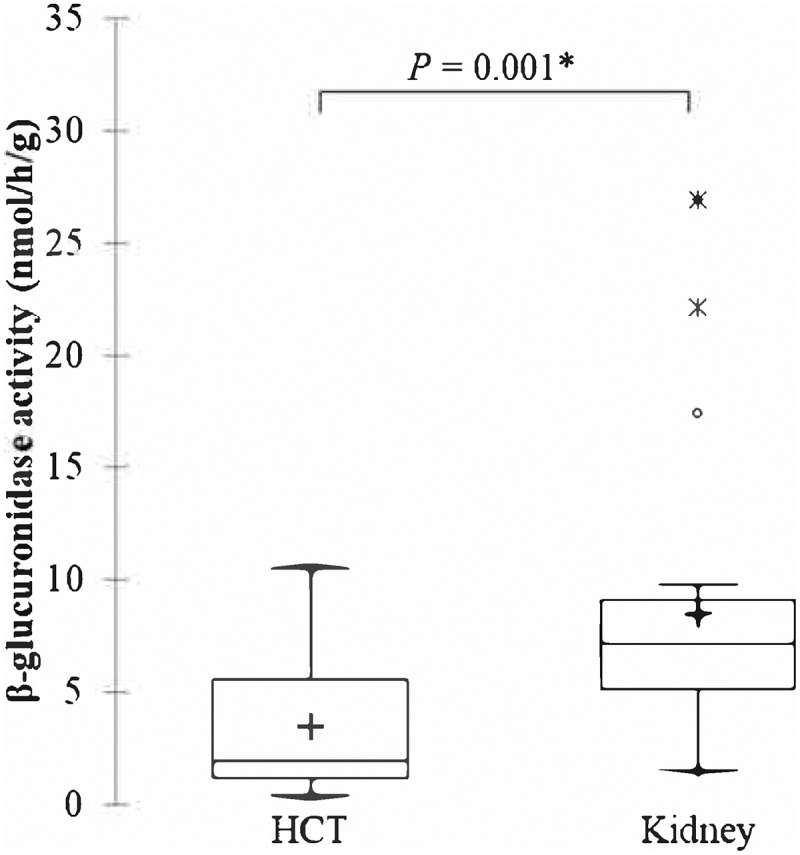
**Β-glucuronidase activity observed in fecal samples from patients in the HCT and MISSION studies**. Boxes show median and interquartile ranges, with mean denoted by **+.**

We compared β-glucuronidase activity from stool samples of patients receiving MMF and tacrolimus post-HCT or kidney transplant. We hypothesized that, based on observed differences in therapeutic dosing regimens between transplantation types, β-glucuronidase activity would differ between transplant types. We observed that fecal β-glucuronidase activity was over two-fold lower in HCT patients (3.50 ± 3.29 nmol/h/g) than in kidney transplant patients (8.48 ± 6.21 nmol/h/g, *P* = .001; Figure 1). Previous studies have found an association of distinct intestinal microbiota with clinical outcomes including infectious complications in HCT recipients^19–21^ and dosing of the IS drug tacrolimus (TAC) in kidney transplant recipients.^22^ In addition, greater abundances of the genera *Blautia* and *Enterococcus*, and decreasing abundances of clostridia have been associated with a lower incidence of GVHD-related mortality in HCT patients.^19–21^ In kidney transplant patients, Lee, *et al*.^22^ hypothesized that the abundance of *Faecalibacterium prausnitzii* was associated with a healthy and diverse colon and found a positive correlation between TAC dosage and *F. prausnitzii*. This hypothesis was tested *in vitro* by Guo, *et al*.^23^ with results suggesting involvement of *F. prausnitzii* in metabolism of TAC. Taken together, these results suggest that improved understanding of the role of the microbiota in biotransformation and transporting IS drugs will be critical to inform precision therapy to maximize efficacy.

There have been few studies that have similarly assessed fecal β-glucuronidase activity in human studies, and results are difficult to compare due to the various methodologies reported.^24^ A recent study of patients with celiac disease, non-celiac gluten sensitivity, and healthy controls found similar levels of fecal β-glucuronidase activity in all groups (mean ± standard deviation: 30.0 ± 15.0, 25.9 ± 15.0, and 29.9 ± 18.0 U g^−1^ wet weight, respectively).^25^ Similarly, an earlier study of children with inflammatory bowel disease (IBD), compared to healthy controls, found that while there was a non-significant difference in fecal β-glucuronidase activity between groups, the activity from children with IBD (mean 15.86, range 0.12–81.63 mM phenolphthalein/mg protein/h) was less than half that of controls (44.86, 5.82–141.13 mM/mg/h). In a study of kidney transplantation patients who received MMF, no differences in fecal β-glucuronidase activity were observed among patients who had diarrhea *vs*. those who did not (median 4.6 *vs*. 4.4 mg free phenolphthalein/mg protein/h, *P* = .78).^26^ A significantly greater percentage of diarrheal patients with high β-glucuronidase activity (91%) had a prolonged course (≥7 days) of diarrhea, while only 40% of low β-glucuronidase-activity patients had a prolonged course.^27^ In a murine study, exposure to MMF resulted in an altered microbial community composition with increased β-glucuronidase activity that was ablated when vancomycin was given.^28^ Results of these studies highlight the role of microbial β-glucuronidase in MMF-related toxicity and adverse side effects.

Several factors have been associated with variability in MPA concentrations including kidney function, albumin, weight, diabetes and time post-transplant, although they have not been consistently observed in all studies.^17,29,30^ There are also several important drug interactions with MPA. The combination of TAC with MPA results in higher MPA plasma concentrations and all of our patients received TAC. Antibiotics such as norfloxacin and metronidazole, ciprofloxacin, amoxicillin-clavulanic acid result in significantly lower MPA concentrations.^31–33^ These interactions have been hypothesized to be due to antimicrobial-related alterations in gut microbiota that change enterohepatic recirculation and reabsorption of MPA. HCT patients receive chemotherapy and more anti-infective agents than kidney transplant recipients in the early post-transplant period. Our observation that β-glucuronidase activity is significantly lower in HCT recipients is highly consistent with the consequences of high antibacterial drug pressure and elimination of β-glucuronidase producing bacteria.

In our HCT population, 20 adult (18–75 years) participants undergoing allogeneic transplant for hematologic malignancies were enrolled and are described in our previous study.^4^ The HCT study protocol was IRB approved (Study#00005621) and all patients provided written, informed consent. All HCT participants received prophylactic MMF (Cellcept or generic) and TAC (Prograf or generic) at time of stool collection on day +7. Mycophenolate mofetil was administered 1 g every 8 h intravenously (IV) over 2 h, every 8 hours beginning on day +5. Tacrolimus (0.03 mg/kg/day) was administered IV beginning at post-transplant day 5 to promote stem cell engraftment and to prevent GVHD. In our kidney transplant study, patients were enrolled in the Microbiome and Immunosuppression (MISSION) study (NCT04953715). This study was IRB approved (Study#00032309) and patients provided written, informed consent. Twenty-two adult patients (≥18 years) were studied and the stool collected a median of 69 days of receiving a living or deceased donor kidney transplant. Patients were receiving MMF (Cellcept or generic) 500 mg/g twice a day and TAC (Prograf or generic) orally as maintenance IS.

Stool samples were collected in single-use specimen collector pans and transferred to 30 mL polystyrene tubes for storage. Single samples were collected from each patient. Samples were refrigerated immediately and transferred to the lab frozen (−20°C) and stored at −80°C prior to assay. Samples from patients in both the HCT and kidney-transplantation studies were treated identically for lab analysis. β-glucuronidase activity was measured in stool samples using a fluorometric assay kit (ab234625; Abcam, Cambridge, United Kingdom) according to the manufacturer’s instructions. Briefly, 100 µl of β-glucuronidase assay buffer (BGA) was added to a 10 mg (wet weight) stool sample. The sample was homogenized at room temperature for 10 min using an ultrasonic bath (2 A, 40 kHz constant frequency; Thermo Fisher Scientific, Waltham, MA, United States). The lysates were then centrifuged at 10,000 × *g* for 5 min at 4°C. The supernatant volume was adjusted to 90 µl with BGA before adding 10 µl of proprietary substrate (provided with the ab234625 kit). All reactions were performed in Microfluor™ 96-well black plates with flat bottoms (Thermo Fisher Scientific). Fluorescence measurement (Ex/Em = 330/450 nm) was done on a Biotek ELISA reader (Agilent Technologies, Santa Clara, CA, United States) immediately after adding substrate for 0–60 mins at 37°C. A background (no substrate) control, positive control and standard (4-methylumberlliferone; 4-MU) dilutions were also included while performing the assay. Enzyme activity was expressed as nmol 4-MU hr^−1^ gm^−1^ sample. Differences in β-glucuronidase activity were evaluated using the non-parametric Kruskal Wallis test. Statistical analyses were performed using XLSTAT software version 2020.3.1 (Addinsoft, Belmont, MA) at *α* = 0.05.

## Data Availability

The authors confirm that the data supporting the findings of this study are available within the article.
